# Metabolic Control by S6 Kinases Depends on Dietary Lipids

**DOI:** 10.1371/journal.pone.0032631

**Published:** 2012-03-07

**Authors:** Tamara R. Castañeda, William Abplanalp, Sung Hee Um, Paul T. Pfluger, Brigitte Schrott, Kimberly Brown, Erin Grant, Larissa Carnevalli, Stephen C. Benoit, Donald A. Morgan, Dean Gilham, David Y. Hui, Kamal Rahmouni, George Thomas, Sara C. Kozma, Deborah J. Clegg, Matthias H. Tschöp

**Affiliations:** 1 Institute for Clinical Biochemistry and Pathobiochemistry, German Diabetes Center, Düsseldorf, Germany; 2 Metabolic Disease Institute, Division of Endocrinology, Department of Medicine, University of Cincinnati College of Medicine, Cincinnati, Ohio, United States of America; 3 Department of Molecular Oncogenesis, Metabolic Disease Institute, University of Cincinnati, Cincinnati, Ohio, United States of America; 4 Department of Molecular Cell Biology, Sungkyunkwan University School of Medicine, Seoul, South Korea; 5 HelmholtzZentrum München, German Research Center for Environmental Health (GmbH), Neuherberg/Munich, Germany; 6 Heidelberg Institute for Stem Cell Technology and Experimental Medicine (HI-STEM), Germany Division of Stem Cells and Cancer, German Cancer Research Center (DKFZ), Heidelberg, Germany; 7 Department of Internal Medicine, College of Medicine, University of Iowa, Iowa City, Iowa, United States of America; 8 Resverlogix Corporation (TSX:RVX), NW Calgary, Alberta, Canada; 9 Department of Pathology, Centre of Arteriosclerosis Studies, Metabolic Disease Institute, University of Cincinnati, Cincinnati, Ohio, United States of America; 10 Department of Internal Medicine, University of Texas Southwestern Medical Center, Dallas, Texas, United States of America; University of Córdoba, Spain

## Abstract

Targeted deletion of S6 kinase (S6K) 1 in mice leads to higher energy expenditure and improved glucose metabolism. However, the molecular mechanisms controlling these effects remain to be fully elucidated. Here, we analyze the potential role of dietary lipids in regulating the mTORC1/S6K system. Analysis of S6K phosphorylation in vivo and in vitro showed that dietary lipids activate S6K, and this effect is not dependent upon amino acids. Comparison of male mice lacking S6K1 and 2 (S6K-dko) with wt controls showed that S6K-dko mice are protected against obesity and glucose intolerance induced by a high-fat diet. S6K-dko mice fed a high-fat diet had increased energy expenditure, improved glucose tolerance, lower fat mass gain, and changes in markers of lipid metabolism. Importantly, however, these metabolic phenotypes were dependent upon dietary lipids, with no such effects observed in S6K-dko mice fed a fat-free diet. These changes appear to be mediated via modulation of cellular metabolism in skeletal muscle, as shown by the expression of genes involved in energy metabolism. Taken together, our results suggest that the metabolic functions of S6K in vivo play a key role as a molecular interface connecting dietary lipids to the endogenous control of energy metabolism.

## Introduction

Control of energy balance plays a central role in diseases such as obesity and metabolic syndrome, where pharmacological suppression of appetite alone appears to be insufficient to prevent or reverse weight gain and adiposity. In order to control the balance between food intake (FI) and energy expenditure (EE), however, further insights will be required into the molecular pathways that control key processes such as nutrient sensing and energy metabolism, and in particular the regulatory mechanisms that integrate them.

S6 kinases (S6K) 1 and 2 are key effectors of the mammalian target of rapamycin complex 1 (mTORC1) and are well known to play a positive anabolic role in insulin-mediated cell growth and to suppress insulin signaling under conditions of hyperactivation of the mTORC1 pathway [1]. The mTORC1/S6K pathway has also been implicated in nutrient sensing, however. Glucose and leucine are known to function as key dietary components involved in activating S6K [2], [3], and dietary fatty acids are also known to activate S6K in liver and muscle [4] and in the hypothalamus [5] of rats fed on a high-fat diet (HFD).

Apart from a potential role in nutrient sensing, S6K1 has also been reported to control systemic energy homeostasis. We have previously demonstrated that global S6K1 deficiency leads to higher EE and a lean phenotype, even when mice are chronically exposed to a HFD with a 60% lipid content [2]. In contrast, S6K over-activation in the hypothalamus decreases EE [6]. The mechanism through which S6K controls energy homeostasis could involve regulation of lipid metabolism, since ex vivo studies in muscle, primary hepatocytes and epididymal white adipose tissue have shown that the combined absence of S6K1 and 2 leads to changes in AMPK activity, mitochondrial biogenesis and beta oxidation [7]. However, it remains unclear whether these effects are retained in vivo or whether the control of systemic energy metabolism by S6K is regulated by dietary lipids.

To further elucidate the molecular mechanisms underlying systemic regulation of energy balance by the mTORC1/S6K pathway, we assessed the role of dietary lipids in determining the metabolic phenotype of mice lacking S6K 1 and 2. The results of this study indicate that the metabolic control of energy balance by S6K is regulated by dietary lipids.

## Results

### Fatty acids activate S6K1 in vitro independently of branched-chain amino acids

**Figure 1:**
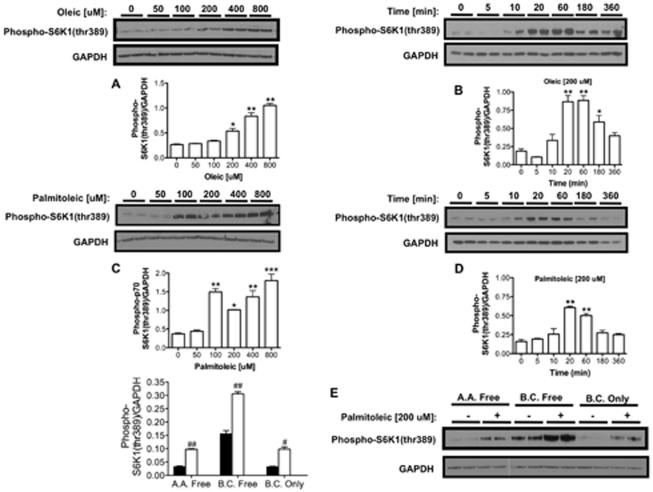
Fatty acids activate S6K1 in vitro in the muscle cell line Sol 8. Oleic acid increased S6K1 phosphorylation in Sol 8 cells in a dose-dependent (A) and time-dependent (B) manner. Similar results were obtained with palmitoleic acid (C and D). S6K1 phosphorylation induced by palmitoleic acid was independent of branched-chain amino acids and amino acids in general (E). A.A., amino acid; B.C., branched-chain amino acid. One way ANOVA *P<0.05, **P<0.01, ***P<0.001; two-tailed *t* test #*P*<0.05, ##*P*<0.01.

Earlier experiments in rats suggested that exposure to a HFD might activate S6K1 in muscle, liver and mediobasal hypothalamus [4], [5]. We therefore analyzed whether relevant dietary fatty acids such as oleic acid and palmitoleic acid can directly induce S6K phosphorylation in vitro. Incubation of muscle Sol 8 cells or neuronal N-41 cells with oleic acid, palmitoleic acid or linoleic acid consistently activated S6K1 in a dose- and time-dependent manner (Fig. 1A–D and Figs. S1A–B, S3).

Since S6K1 has been reported to function in vivo as a nutrient sensor that responds to branched-chain amino acids such as leucine [3], we further assessed whether S6K1 activation by fatty acids was dependent on amino acids. Both oleic acid and palmitoleic acid induced S6K1 phosphorylation independently of branched-chain amino acids or indeed any other amino acids (Fig. 1E and Fig. S1C). The S6K1 phosphorylation induced by these fatty acids was enhanced by insulin and prevented by rapamycin, as expected for an effector pathway downstream of mTORC1 (Fig. S1D).

### Dietary NEFAs activate skeletal muscle S6K in vivo

**Figure 2:**
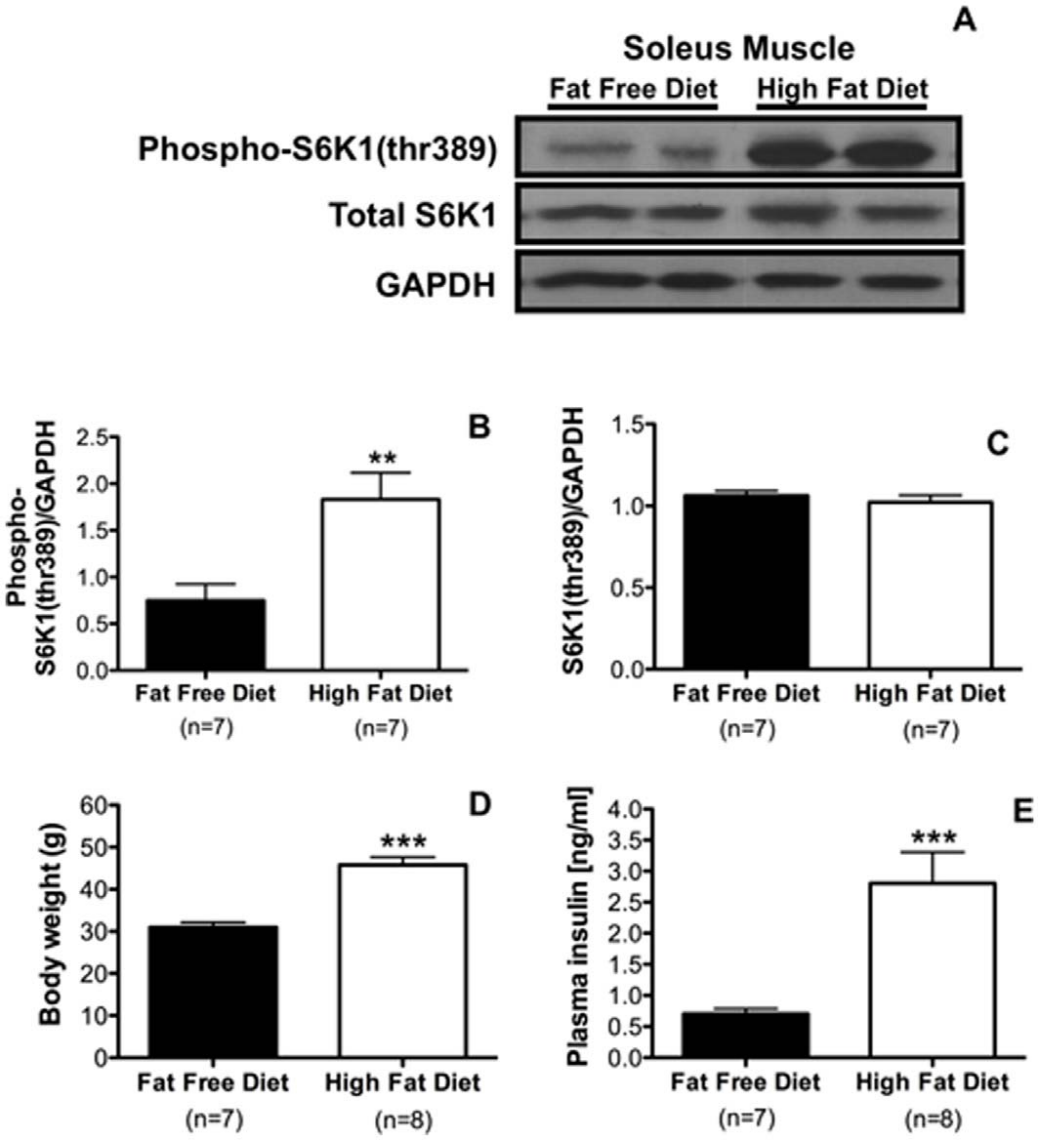
Dietary fatty acids activate S6K1 in muscle. Mice fed a high-fat diet for 6 wk show significantly higher phosphorylated S6K1 (A, B) with no changes in total S6K1 protein levels when compared to control mice fed a fat-free diet (C). Body weight (D) and plasma insulin levels (E) were significantly higher in mice on a high-fat diet. Two tailed *t* test **P<0.01, ***P<0.001.

Consistent with our in vitro findings, male C57BL/6 mice reared on a HFD (60% fat content, n = 7) for 6 wk showed a significantly higher level of phosphorylated S6K in muscle (*P*<0.01) compared to those that received a FFD (0% fat content, n = 7) (Fig. 2A, B) with no differences in total S6K1 protein levels (Fig. 2C). As expected, mice fed a HFD (n = 8) showed significantly increased bodyweight (45.77±1.85 g, *P*<0.001) and plasma insulin (2.81±0.5 ng/ml, *P*<0.001) compared to the FFD control group (30.98±1.16 g and 0.71±0.08 ng/ml, respectively; n = 7) (Fig. 2D, E).

### Targeted deletion of S6K protects against HFD-induced obesity

**Figure 3:**
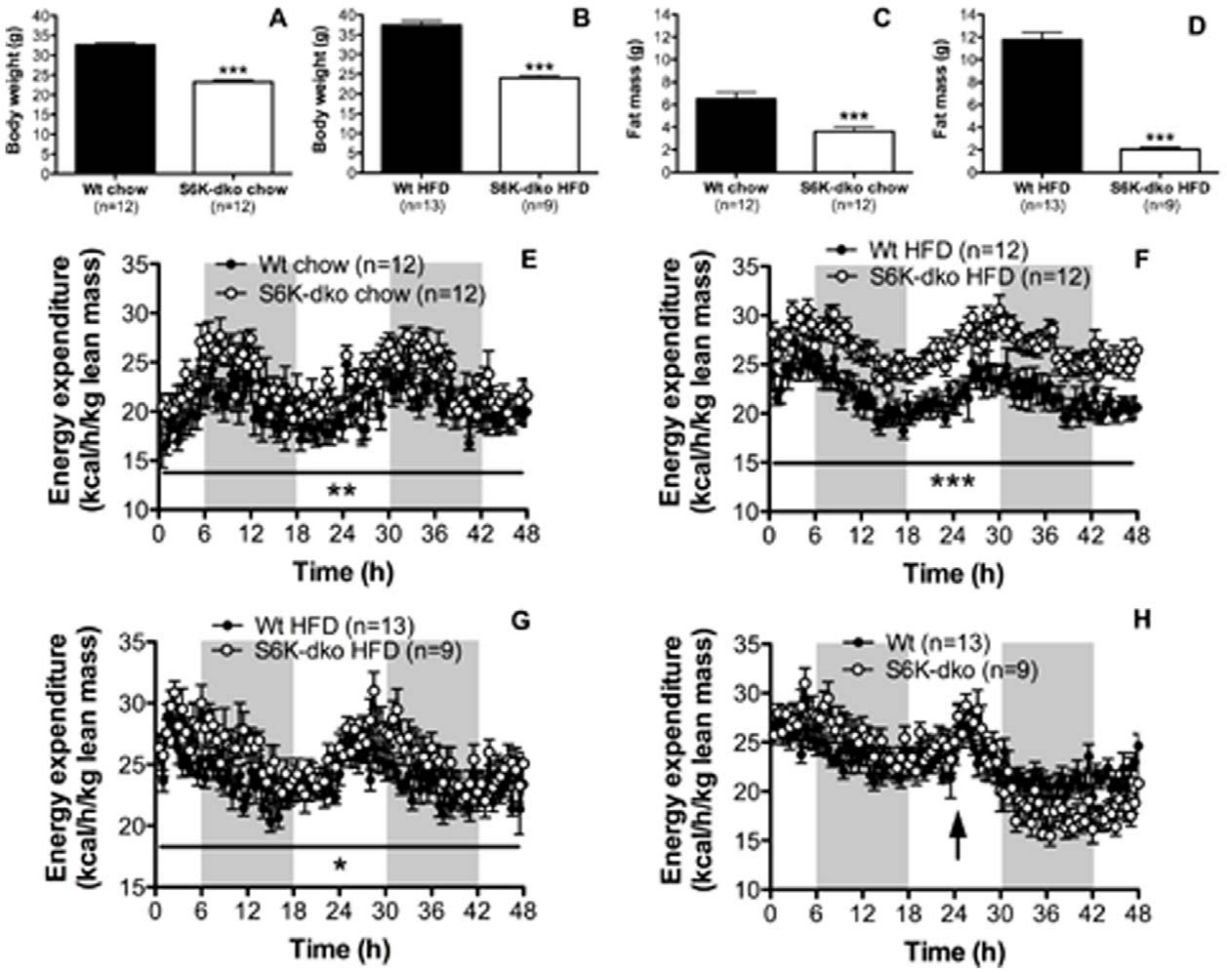
S6K-dko have higher energy expenditure levels. S6K-dko have significantly lower body weight (A) and fat mass (C) than wt controls even when fed a high-fat diet (B, D). Energy expenditure corrected by lean mass levels is higher in S6K-dko when fed standard chow (E), acute high-fat diet (F) or chronic high-fat diet (G) compared to wt controls. Higher energy expenditure in S6K-dko is reduced with 24 h fasting (H). HFD, high-fat diet. Two-tailed *t* test ***P<0.001 and two-way ANOVA *P<0.05, **P<0.01, ***P<0.001.

To uncover the functional relevance of in vivo S6K activation by dietary lipids, we compared the effect of HFD and standard chow diet in S6K-dko and wt mice. S6K-dko mice had a significantly lower BW (Fig. 3A, B) and fat mass (Fig. 3C, D) on both standard chow diet (23.30±0.45 g and 3.63±0.39 g, respectively; n = 12; *P*<0.001) and HFD (24.03±0.57 g and 2.04±0.20 g, respectively; n = 9; *P*<0.001) compared to wt controls (standard chow, 32.59±0.51 g and 6.51±0.57 g, respectively; HFD, 37.39±1.1 g and 11.75±0.67 g, respectively; n = 12–13). S6K-dko mice had persistent lower BW than wt mice despite having a higher FI on both standard chow and HFD (Fig. S2A, S2B). Consistent with this observation, EE measured for 48 h and normalized to lean mass was significantly higher in S6K-dko (n = 12, *P*<0.01) than wt control mice (n = 12) when both were fed on standard chow (Fig. 3E). This difference in EE was amplified considerably upon acute exposure (5 d) to a HFD containing 60% lipids (n = 12, *P*<0.001) (Fig. 3F). The EE levels remained significantly higher in S6K-dko on a HFD for more than 9 mo (n = 9, *P*<0.05) when compared to age- and sex-matched wt controls reared under similar conditions (n = 13) (Fig. 3G).

### The metabolic phenotype of S6K-dko mice depends on dietary lipids

**Figure 4:**
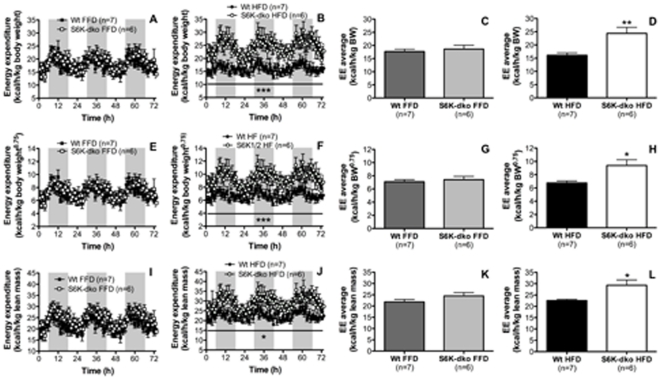
Energy expenditure levels in S6K-dko are diet regulated. Energy expenditure normalized for body weight (B, D), body surface area (F, H) or lean mass (J, L) is higher in S6K-dko than wt mice when fed a high-fat diet. No differences in energy expenditure are observed between S6K-dko and wt control mice fed a fat-free diet (A, C, E, G, I, K). BW, body weight; EE, energy expenditure; FFD, fat-free diet; HFD, high-fat diet. Two-tailed *t* test *P<0.05, **P<0.01 and two-way ANOVA *P<0.05, ***P<0.001.

To address the role of dietary macronutrients in the control of energy metabolism by S6K in vivo, we first analyzed EE in S6K-dko mice that had been fasted for 24 h (indicated with an arrow in Fig. 3H). EE decreased rapidly in S6K-dko (n = 9) to match that of wt controls (n = 13, Fig. 3H). To further analyze whether the metabolic phenotype of S6K-dko mice was limited to dietary fatty acids, we compared the impact of chronic exposure to HFD versus FFD in a new group of age- and sex-matched wt and S6K-dko mice. Caloric content from protein was kept equal between both diets (20%). S6K-dko mice fed a HFD for 3 mo (n = 6) had significantly higher EE than wt controls (n = 7) when normalized for BW (*P*<0.001, Fig. 4B; 24.4±2.26 kcal/h/kg BW *P*<0.01, Fig. 4D), body surface area (*P*<0.001, Fig 4F; 9.4±0.9 kcal/h/kg BW^0.75^, *P* = 0.0108, Fig 4H) or lean mass (*P*<0.05, Fig. 4J; 29.3±2.34 kcal/h/kg lean mass, *P* = 0.0118, Fig. 4L) compared to the wt control group (16.11±0.9 kcal/h/kg BW, 6.75±0.3 kcal/h/kg BW^0.75^, and 22.56±0.56 kcal/h/kg lean mass). In the absence of dietary lipids (S6K-dko and wt mice on FFD), however, no EE phenotype was detectable (Fig. 4A, C, E, G, I, K).

### Lower fat mass gain and plasma levels of leptin, cholesterol and triglycerides depend on dietary lipids in S6K-dko mice

**Figure 5:**
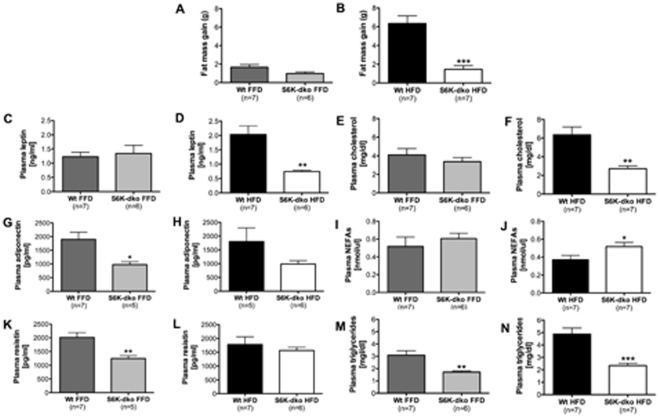
S6K-dko plasma biochemistry. S6K-dko mice fed a high-fat diet show significantly lower fat mass gain (B), plasma leptin (D), cholesterol (F) and triglycerides (N), as well as higher free fatty acids (J) compared to wt controls. When fed a fat-free diet, S6K-dko mice still show significantly lower plasma triglycerides (N), as well as significantly lower adiponectin (H) and resistin (K) levels. FFD, fat-free diet; HFD, high-fat diet; NEFAs, non-esterified fatty acids. Two-tailed *t* test *P<0.05, **P<0.01, ***P<0.001.

S6K-dko mice fed a HFD for 3 mo had significantly lower fat mass gain (1.5±0.4 g, *P*<0.001, n = 7) (Fig. 5B), plasma levels of leptin (0.74±0.05 ng/ml, *P*<0.01, n = 6) (Fig. 5D), cholesterol (2.71±0.31 mg/dl, *P*<0.01, n = 7) (Fig. 5F), and triglycerides (2.33±0.2 mg/dl, *P*<0.001, n = 7) (Fig. 5N), and higher plasma levels of NEFAs (0.52±0.05 nmol/µl, *P*<0.05, n = 7) (Fig. 5J) compared with age- and sex-matched wt control mice (fat mass gain, 6.35±0.8 g; leptin, 2.03±0.30 ng/ml; cholesterol, 6.36±0.83 mg/dl; triglycerides, 4.9±0.49 mg/dl; NEFAs, 0.37±0.05 nmol/µl; n = 7). There were no significant differences in plasma adiponectin or resistin levels between S6K-dko mice (n = 6) and wt controls (n = 5–7) (Fig. 5H, L).

When the mice were fed a FFD, the only identifiable difference was lower plasma adiponectin (977±104 pg/ml, *P*<0.05, n = 5) (Fig. 5G), resistin (1243±106 pg/ml, *P*<0.01, n = 5) (Fig. 5K) and triglycerides (1.72±0.1 mg/dl, *P*<0.01, n = 6) (Fig. 5M) in S6K-dko compared to wt controls (adiponectin, 1897±260 pg/ml; resistin, 2013±178 pg/ml; triglycerides, 3087±0.36 mg/dl; n = 7).

### S6K deficiency protects against HFD-induced glucose intolerance

**Figure 6:**
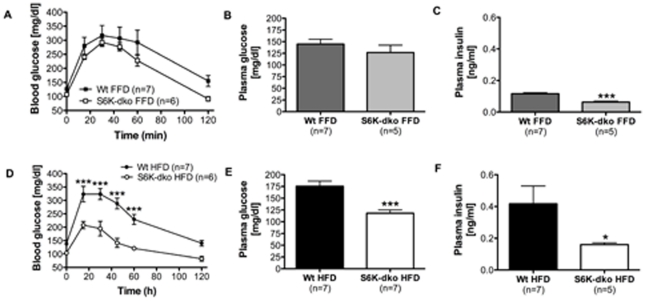
S6K-dko glucose tolerance and diet. S6K-dko mice fed a high-fat diet have significantly higher glucose tolerance (D) and lower plasma glucose (E) and insulin (F) compared to wt controls. When fed a fat-free diet, S6K-dko mice do not show better glucose tolerance (A) or lower plasma glucose levels (B) in spite of having significantly lower plasma insulin levels (C). FFD, fat-free diet; HFD, high-fat diet. One-tailed *t* test *P<0.05, two-tailed *t* test ***P<0.001 and two-way ANOVA ***P<0.001.

S6K-dko mice fed a HFD for 3 mo exhibited greater glucose tolerance (*P*<0.001, n = 6) (Fig. 6D) and significantly lower fasting plasma glucose (118.3±6.8 mg/dl, *P*<0.001, n = 7) (Fig. 6E) and insulin (0.1599±0.01 mg/dl, *P*<0.05, n = 5) (Fig. 6F) compared to wt controls (175.5±10.9 mg/dl and 0.4167±0.11 mg/dl, respectively; n = 7). No such differences in glucose tolerance (Fig. 6A) or fasting plasma glucose (Fig. 6B) were detected when mice were fed a FFD. However, S6K-dko mice had significantly lower plasma insulin (0.0633±0.001 ng/ml, *P*<0.001, n = 5) (Fig. 6C) compared to wt controls (0.1161±0.007 ng/ml, n = 7) when dietary lipids were absent.

### Enhanced muscle fatty acid metabolism in S6K-dko mice depends on dietary lipids

**Figure 7:**
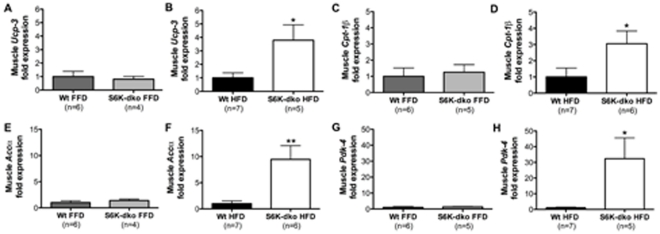
Muscle lipid beta oxidation in S6K-dko is diet regulated. S6K-dko mice fed a high-fat diet have higher muscle lipid beta oxidation, as shown by significantly higher mRNA levels of *Ucp-3* (B), *Cpt-1β* (D), *Accα* (F) and *Pdk-4* (H). No such differences were observed in mice fed a fat free diet (A, C, E, G). FFD, fat-free diet; HFD, high-fat diet. Two-tailed *t* test *P<0.05, **P<0.01.

Muscle tissue from S6K-dko mice fed on a HFD had significantly higher levels of mRNA for *Ucp-3* (*P*<0.05, n = 5) (Fig. 7B), *Cpt-1β* (*P*<0.05, n = 6) (Fig. 7D), *Accα* (*P*<0.01, n = 6) (Fig. 7F) and *Pdk-4* (*P*<0.05, n = 5) (Fig. 7H) compared to muscle from wt controls (n = 6–7). In the absence of dietary lipids, however, no such differences were detected (Fig. 7A, C, E, G).

### BAT thermogenesis does not regulate the dietary lipid-dependent metabolic phenotype of S6K-dko mice

**Figure 8:**
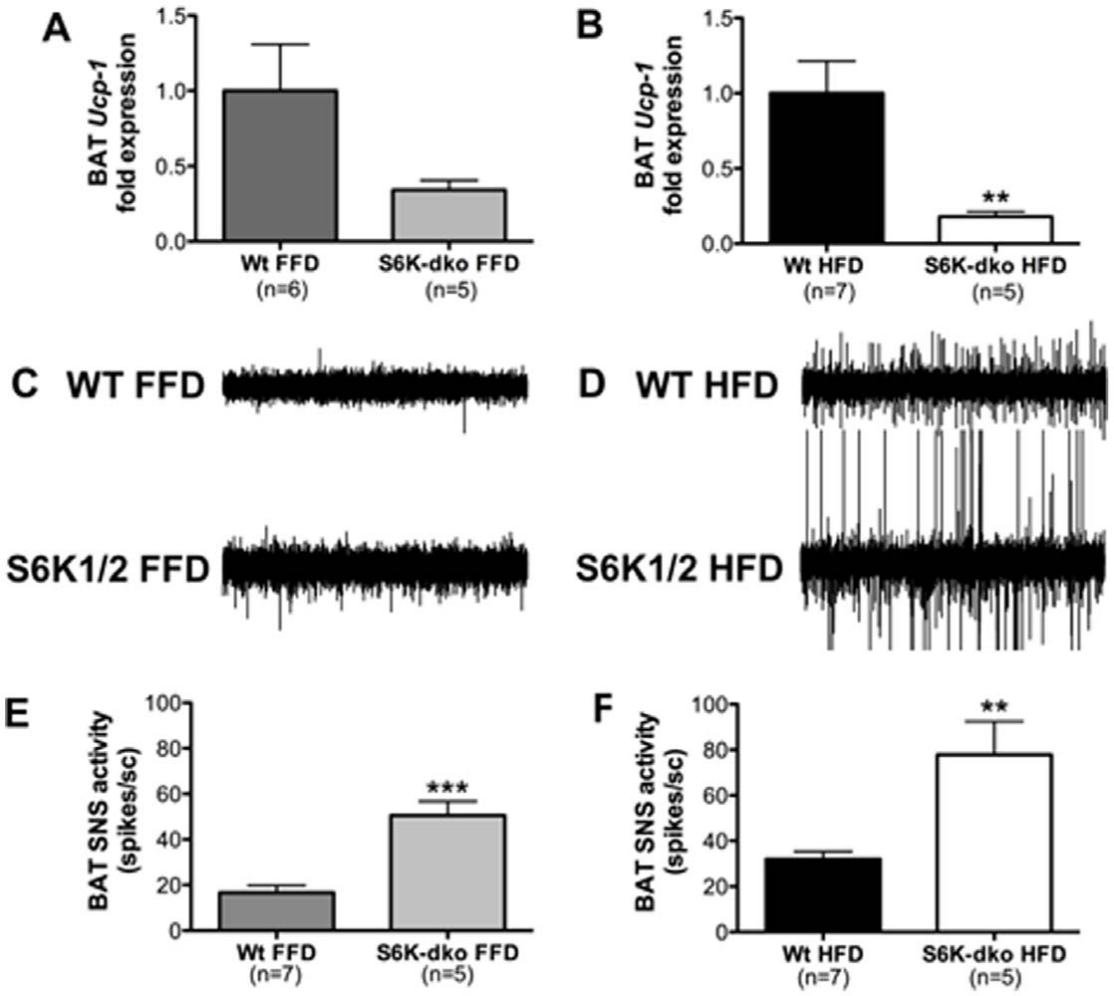
Brown adipose tissue thermogenesis in S6K-dko is diet independent. S6K-dko mice fed a high-fat diet have significantly lower levels of *Ucp-1* mRNA in brown adipose tissue (B) and higher sympathetic activity (D, F) compared to wt controls. When fed a fat-free diet, S6K-dko mice show no significant differences in *Ucp-1* mRNA levels in brown adipose tissue (A) but do have significantly higher sympathetic activity (C, E) compared to wt controls. BAT, brown adipose tissue; FFD, fat-free diet; HFD, high-fat diet. Two-tailed *t* test **P<0.01, ***P<0.001.

BAT from S6K-dko mice (n = 6) fed a HFD for 5 mo had lower *Ucp-1* mRNA levels (n = 5, *P*<0.05) (Fig. 8B) and higher sympathetic activity (n = 6, *P*<0.01) (Fig. 8D, F) compared with BAT from wt controls (n = 7). Therefore, it is unlikely that differences in BAT uncoupling explain the observed energy metabolism phenotype. On a FFD, no significant differences in *Ucp-1* mRNA levels were detected in BAT from S6K-dko mice (n = 5) (Fig. 8A), compared to wt controls (n = 6–7). BAT sympathetic activity in mice fed a FFD (n = 5, *P*<0.001) was still higher in the S6K-dko mice than wt controls (Fig. 8C, E).

## Discussion

The results of this study show that S6Ks are directly activated by fatty acids in vitro and in vivo, a finding that appears to expand the role of S6K as a nutrient sensor. More importantly, our data suggest that the predominant role of S6K in metabolic regulation is in the response to dietary lipids.

Previously, it has been demonstrated that exposure to a HFD leads to the activation of S6K1 in muscle and liver tissue [4] and also in the hypothalamus [5]. Phosphorylation of S6 (a signaling protein downstream of mTORC1 and S6K1 and 2) has also been observed in the hypothalamus of fed ob/ob and db/db mice [8], a finding that is consistent with leptin-independent, nutrient-activated regulation of this pathway. Based on these studies, and our own in vitro and in vivo observations indicating that fatty acids can directly activate S6K1, we hypothesized that the metabolic phenotype of S6K1 and 2 deficient mice is at least partly dependent on exposure to dietary lipids.

When S6K-dko mice were fed a HFD for an extended period, they gained less body fat and exhibited a marked increase in EE compared with wt mice and S6K-dko mice fed on a FFD. S6K-dko mice also had lower fasting leptin levels, lower cholesterol, higher NEFAs and better glucose tolerance on a HFD compared to wt mice. However, none of these features occurred in the absence of dietary lipids. Under these conditions, all mice had normal FI, BW and good health, and long-term FFD exposure did not appear to have any non-specific or adverse effects. Since the metabolic phenotype of S6K-dko mice—involving changes in adiposity, energy metabolism, and glucose tolerance—fails to develop when the mice are fed on a FFD, we conclude that metabolic control by S6K is predominately regulated by dietary lipids.

Hypothalamic S6K over-activation in rats decreases EE [6], whereas S6K1-ko and adipose tissue-specific raptor ko mice show higher EE levels [2], [9], [10]. Consistent with the results of those studies, S6K-dko mice show high EE. Defining the specific tissue responsible for the considerable EE phenotype is of great interest. Our results indicate that BAT is unlikely to be the only cause of the significantly higher EE levels in response to lipids, since *Ucp-1* mRNA expression in S6K-dko mice fed a HFD was lower despite higher BAT sympathetic activity. In fact, adipose tissue has been proposed as a source of higher levels of EE in mice with specific deletion of raptor in this tissue [9]. Consistent with our results, expression of uncoupling proteins was similar to or lower than in controls in that study. On the other hand, we found that the expression of genes linked to energy metabolism, including *Ucp-3*, *Cpt-1β*, *Accα* and *Pdk-4*, was up-regulated in skeletal muscle when S6K-dko mice were fed a HFD. This suggests that skeletal muscle thermogenesis is a more likely cause of the substantial EE response to dietary lipids. This finding is consistent with recently published data in which the absence of S6K or treatment with rapamycin promoted fatty acid oxidation in muscle cells [7], [11]. Interestingly, mice lacking raptor in skeletal muscle die early and show muscle dystrophy, lower muscle mitochondrial biogenesis and reduced oxidative capacity [12].

The physiological events associated with the absence of S6K resemble those seen with caloric deprivation, in which insulin levels are low and gluconeogenesis is augmented. In both situations, lipids are used as the main source of energy in order to preserve glucose for the central nervous system and erythrocytes [13], [14]. The significantly higher fasting NEFAs and lower plasma triglyceride levels observed in S6K-dko fed a HFD could suggest a higher sensitivity and more efficient response to the perceived lack of dietary lipids. Consistent with this possibility, rapamycin administration in human lymphoma cells alters gene expression in a manner similar to that observed upon starvation [15] and decreased mTOR signaling has been linked to starvation-induced stress, higher mitochondrial respiration and extended life-span in yeast [16], [17], [18]. In mammals, mTOR signaling has also been linked to longevity [19]. Specifically, levels of phosphorylated S6K1 were reduced in muscle and liver in a mouse model of extended life span [20]. However, it remains possible that the physiological differences observed in our study are also a consequence of altered sensing of dietary sugars, as the HFD and FFD used differed in carbohydrate content, and skeletal muscle energy metabolism depends heavily on insulin-stimulated glucose uptake [21].

We found that S6K activation by fatty acids in vitro depends on mTORC1 but not on amino acids. However, the mechanisms underlying this observation remain unclear. mTORC1 translocation to lysosomal membranes and subsequent activation in vitro occurs in response to amino acids [22], [23]. However, it is not clear how NEFAs influence this pathway in similar cell models (human embryonic kidney cells and mouse embryonic fibroblasts). Further studies will therefore be required to elucidate the mechanisms underlying fatty acid-dependent, amino acid-independent mTORC1/S6K activation and function.

Since exposure to dietary fatty acids may directly affect nutrient sensing neurons expressing S6K in the arcuate nucleus in vivo [3], the mediobasal hypothalamus could represent an important site at which the action of S6K links dietary lipids to the control of adaptive thermogenesis. According to such a model, S6K in the arcuate nucleus could be involved in the regulation of skeletal muscle metabolism by the central nervous system. However, tissue-specific gene targeting will be necessary to address whether these differences in energy metabolism in response to dietary lipids are a direct consequence of the lack of S6K activation in skeletal muscle, an indirect effect of S6K modulation in the central nervous system influencing muscle EE via efferent SNS activity, or a combination of the two.

An intriguing effect of null mutations for both S6Ks is the protection against lipotoxicity [24], presumably as a collateral effect of the enhanced muscle fatty acid beta oxidation in mice fed a HFD. Since the mTORC1 pathway mediates cell proliferation [25], as reflected by the smaller size of the S6K-dko mice [26], and lack of S6K1 impairs adipogenesis [27], lipid deposition outside of adipose tissue might be expected in response to chronic HFD exposure. However, higher lipid oxidation in skeletal muscle apparently provides protection against such adverse effects and thereby prevents the detrimental metabolic consequences of limited adipose tissue expansion capabilities in the absence of S6K. This suggests that the absence of S6K not only protects against obesity but also against potential lipotoxicity as a result of collateral hypotrophy.

**Figure 9:**
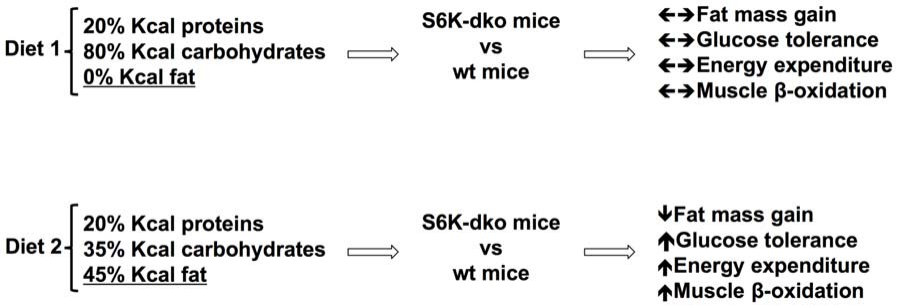
Model of metabolism regulation by diet in absence of S6K. Mice S6K-dko show lower fat mass gain and higher glucose tolerance, energy expenditure and muscle lipid β-oxidation compared with wt mice when consuming a high fat diet. Those metabolic differences are not present in absence of dietary lipids.

In summary, we conclude that S6K deficiency in the absence of dietary lipids has no physiologically relevant metabolic consequences even when animals are chronically exposed to considerable amounts of dietary carbohydrates (Fig. 9). Our findings are consistent with a role for S6K activity in the development of dietary fatty acid-induced metabolic disease. Finally, our results show that S6K deficiency specifically protects against the pathological effects of dietary lipids and suggest that the S6K pathway in skeletal muscle may represent a valuable molecular target for the treatment of metabolic diseases. Further studies will be necessary to elucidate the role of S6Ks in skeletal muscle and central nervous system as a putative molecular interface between dietary lipids and the endogenous control of systemic energy and glucose and lipid metabolism.

## Materials and Methods

### Animals

Mice lacking *S6K1* and *S6K2* (S6K-dko) were generated as described by Pende at al. [26]. Age- and sex-matched S6K1-ko mice were originally generated in a mixed 129/SveJ×C57Bl/6 line and the S6K2-ko mice in a mixed 129/OlaHSD×C57Bl/6 line [26], [28]. Male S6K-dko and wild-type (wt) mice were housed in individual cages under a 12∶12 h light-dark cycle (7 am light on) with ad libitum access to standard chow (6% kcal from fat, Harlan Teklad Laboratory Diets, Wisconsin, USA), fat-free diet (FFD) (0% kcal from fat, D04112303 Research Diets Inc., New Jersey, USA) or HFD (60% kcal from fat [D12492] and 45% kcal from fat [D12451], Research Diets Inc., New Jersey, USA) and tap water. All animal protocols were approved by the University of Cincinnati Institutional Animal Care and Use Committee. For the first round of experiments, mice received standard chow or 60% kcal HFD for 9 mo after weaning; for the second round of experiments, mice were placed on FFD or 45% HFD at 2 mo and continued to receive that diet for 3–5 mo.

### Energy balance measurements

Mice were placed in a customized indirect calorimetry system (TSE Systems GmbH, Bad Homburg, Germany) and FI and EE were monitored simultaneously for 2–3 d after adaptation. FI measurements were taken every 30 min and EE measurements were taken every 45 min at al flow rate of 0.5 l/min. Body composition measurements were taken using nuclear magnetic resonance (Whole Body Composition Analyzer; Echo MRI, Texas, USA).

### Glucose tolerance test

Analysis of glucose tolerance was carried out in mice fasted overnight and injected intraperitoneally with 2 g glucose per kg body weight (BW) (50% wt/vol D-glucose [Sigma, St Louis, MO, USA] in 0.9% wt/vol NaCl). Tail-blood glucose levels (mg/dl) were measured with a glucometer (TheraSense Freestyle) and standard glucose strips (Free Style; Abbott Laboratories) before (0 min) and at 15, 30, 45, 60 and 120 min after injection.

### Plasma analysis

Mice were killed by decapitation at the end of the study and blood was collected and immediately chilled on ice. Plasma was obtained by centrifugation at 3000 *g* and 4°C for 15 min and then stored at −80°C. Non-esterified fatty acid (NEFA) levels were measured using a commercially available enzymatic assay kit (Autokit NEFA C, Wako, Neuss, Germany). Plasma cholesterol and triglycerides were determined using Infinity Cholesterol reagent and Infinity Triglyceride reagent b (Thermo Electron, Pittsburgh, Pennsylvania, USA). Plasma glucose was measured using a commercial kit based on the glucose oxidase method (Biomerieux, Marcy l'Etoile, France). Plasma insulin and leptin were measured using a mouse radio-immunoassay (Linco Research, St Charles, MO, USA). The intra- and inter-assay coefficient of variation was 2.7–5.8% and 3.8–10.8%, respectively, for insulin and 4.0–11.2% and 3.3–14.6%, respectively, for leptin. Plasma adiponectin and resistin were measured with a Luminex100 TM IS analyzer (Luminex Corporation, Austin, TX, USA) and mouse lincoplex kits (Linco Research, St Charles, MO, USA). The intra- and inter-assay coefficient of variation was <5% and <12%, respectively, for adiponectin and <4.5% and <10.3%, respectively, for resistin. All assays were performed according to the manufacturer's instructions.

### Real-time quantitative PCR

S6K-dko and wt mice were killed following sympathetic nervous system (SNS) recordings performed after approximately 5 mo of exposure to FFD and HFD. Brown adipose tissue (BAT) and gastrocnemius muscle were collected and stored at −80°C prior to analysis of mRNA expression for uncoupling protein 3 (*Ucp-3*), carnitinepalmitoyl-transferase 1 beta (*Cpt-1ß*), acetyl coenzyme A carboxylase (*Acc*) alpha and pyruvate dehydrogenase kinase 4 (*Pdk-4*) by real-time quantitative PCR (qPCR) in an iCycler Optical Module (BioRad Hercules, CA, USA) (Table 1). Total RNA was extracted from frozen tissue samples using the RNeasy Lipid Mini Kit (Qiagen, Valencia, CA, USA). cDNA templates for qPCR were synthesized using 0.5 µg of total RNA, 10× DNase reaction buffer, DNase 1 amp grade, dNTPs, random primers, and Superscript III reverse transcriptase (Invitrogen, Carlsbad, CA, USA). qPCR was performed using iQ SYBR Green Supermix (BioRad, Hercules, CA, USA) according to the standard protocol with approximately 70 ng template cDNA. All primers were used at a final concentration of 0.5 µM. A standard curve was used to obtain the relative concentration of each experimental gene; values were normalized to the concentration of the housekeeping gene hypoxanthine–guanine phosphoribosyltransferase (*Hprt*) in each sample.

**Table 1:** Primer sequences for RT-PCR.

Mouse gene	Accession #	Forward sequence	Reverse sequence	Product	Annealing T
*Ucp-1*	NM_009463	5′GGGCCCTTGTAAACAACAAA3′	5′GTCGGTCCTTCCTTGGTGTA3′	196	60
*Ucp-3*	NM_009464	5′GTCTGCCTCATCAGGGTGTT3′	5′CCTGGTCCTTACCATGCAGT3′	204	60
*Cpt-1β*	NM_009948	5′CAGCGCTTTGGGAACCACAT3′	5′CACTGCCTCAAGAGCTGTTCTC3′	203	60
*Acc-α*	NM_133360	5′-GCCTCTTCC TGACAAACGAG-3′	5′-TGACTGCCG AAACATCTCTG-3′	239	60
*Pdk4*	NM_013743	5′CCTTTGGCTGGTTTTGGTTA3′	5′CCTGCTTGGGATACACCAGT3′	294	60
*Hprt*	NM_013556	5′TGCTCGAGATGTCATGAAGG3′	5′TATGTCCCCCGTTGACTGAT3′	196	60

### Cell culture and Western blot analysis

The mouse muscle cell line Sol 8 was a generous gift from Dr. Silvana Obici (University of Cincinnati) and the hypothalamic cell line N-41 was obtained from Cellutions Biosystems Inc. (Toronto, Ontario, Canada). Sol 8 and N-41 cells were grown and maintained in high-glucose Dulbecco's Modified Eagle's Medium (DMEM) (Hyclone Laboratories, Logan, UT, USA) containing 20% and 10% fetal bovine serum (FBS) (GIBCO Laboratories, Grand Island, NY, USA), respectively, with antibiotic/antimycotic mix (GIBCO Laboratories, Grand Island, NY, USA) at 37°C in a 5% CO_2_ atmosphere. Differentiation of Sol 8 cells was achieved by seeding the cells in 12-well plates and growing them to 50% confluence before switching the medium to DMEM containing 5% horse serum (Sigma, St Louis, MO, USA) for 3 d. For experiments, Sol 8 and N-41 cells were washed with PBS and incubated in serum-free low-glucose DMEM for 24 h prior to and during experiments. Serum-starved cells were then treated for the specified times with palmitoleic and oleic acid (200 µM) preincubated with 0.1% fatty acid-free BSA overnight at 4°C (Sigma, St Louis, MO, USA). Following the different treatments, the cells were lysed in ice-cold RIPA buffer containing protease inhibitors (1× PBS, 1% Nonidet P40, 0.5% sodium deoxycholate, 0.1% SDS with 50 mM NaF, 0.5 M phenylmethylsulfonyl fluoride, 0.1 mM sodium vanadate, aprotinin [10 µg/ml], and leupeptin [5 µg/ml]) (Sigma, St Louis, MO, USA). N-41 cells were also incubated with or without insulin and the mTOR inhibitor rapamycin (20 nM) (Sigma, St Louis, MO, USA). Following the different treatments, the cells were lysed in ice cold RIPA buffer containing protease inhibitors. Lysates were collected in a 1.5-ml tube and cleared by centrifugation at 4°C for 10 min at 7500 rpm. An aliquot of sample was used for determination of protein concentration. Samples were then boiled in 4× lithium dodecyl sulfate/dithiothreitol buffer (Invitrogen, Carlsbad, CA, USA; American Bioanalytical, Natick, MA, USA) for 10 min at 70°C. Cell lysates (50 µg protein) were loaded on 9% (w/v) acrylamide resolving gels, separated by SDS-PAGE and transferred to Hybond ECL nitrocellulose membranes (Amersham Biosciences, Piscataway, NJ, USA). Membranes were blocked in 2% ECL advance blocking agent for 1 h at room temperature and probed with primary antibodies for S6K (Cell Signaling, Beverly, MA, USA) and glyceraldehyde 3-phosphate dehydrogenase (GAPDH) (Santa Cruz Biotechnology, Santa Cruz, CA, USA). After washing, primary antibodies were detected using either HRP-conjugated anti-mouse IgG for phosphorylated-S6K (Thr389) or anti-rabbit IgG for GAPDH (Bio-Rad, Hercules, CA, USA). Protein bands were visualized using advance chemiluminescence (Amersham Biosciences, Piscataway, NJ, USA), exposed to CL-Xposure film (Pierce, Rockford, IL, USA) and then quantified using AlphaEase FC software.

To measure S6K activation independently of NEFA, Sol 8 and N-41 cells were treated as described above. After 24 h incubation in serum-free medium, cells were washed in PBS and incubated for 2 h in amino acid-free DMEM basic medium. Cells were then treated with BSA-conjugated palmitoleic acid (200 µM) for 1 h in either amino acid-free, branched-chain amino acid-free or branched-chain amino acid-supplemented DMEM. Cells were lysed and treated as previously described.

For quantification of S6K in the soleus muscle of male C57BL/6 mice, both soleus muscles were collected from animals reared on FFD and 60% HFD and euthanized after 6 wk. Tissues were immediately placed in RIPA buffer containing protease inhibitors, and then samples were lysed with a TissueLyser (Qiagen, Valencia, CA, USA) for 3 min and centrifuged at 4°C for 10 min at 7500 rpm. Supernatant was transferred to a new tube and an aliquot was taken for protein quantification. Phosphorylated-S6K1 (Thr389) and total S6K1 were quantified using the protocol described above.

### Sympathetic nervous system recordings

Each mouse was initially anesthetized by intraperitoneal injection with a ketamine/xylazine cocktail (91 mg/kg ketamine and 9.1 mg/kg xylazine). A PE-50 tube was inserted into the trachea for unobstructed spontaneous breathing and a catheter was inserted into the right jugular vein to maintain anesthesia with alpha-chloralose (25 mg/kg initially, followed by 6 mg/kg/h during the protocol). To monitor blood pressure and heart rate, a catheter was inserted into the left carotid artery. Rectal body temperature was maintained at 37.5°C using a lamp and heating pad. A nerve innervating BAT was isolated and placed on bipolar platinum-iridium electrodes for recordings. The nerve signal was amplified 100,000× at high-frequency and low-frequency cutoffs of 1 kHz and 100 Hz, respectively. Blood pressure, heart rate and BAT sympathetic nerve activity were recorded continuously for the next 20–30 min. At the end of the recording session, the nerve was broken and the background noise was measured and subtracted from baseline activity of the BAT SNS.

### Statistical analysis

Quantitative data are presented as mean ± standard error of the mean (SEM). Data were compared by one-way and two-way repeated measures analysis of variance (ANOVA) with post-hoc Bonferroni and Dunnett tests and by one- or two-tailed, unpaired *t* tests. *P*<0.05 was considered statistically significant (GraphPad Prism software).

## Supporting Information

Figure S1 **Fatty acids activate S6K1 in vitro in the hypothalamic cell line N-41.** Palmitoleic acid increases S6K1 phosphorylation in N-41 cells in a dose-dependent (A) and time-dependent (B) manner. Palmitoleic acid-induced phosphorylation was independent of branched-chain amino acids and amino acids in general (C). S6K1 phosphorylation induced by palmitoleic acid was enhanced by insulin and prevented by rapamycin, suggesting a mechanism involving the mTORC1 pathway (D). A.A., amino acid; B.C., branched-chain amino acid. One way ANOVA *P<0.05, **P<0.01, ***P<0.001; two-tailed *t* test #*P<*0.05.
(TIF)

Figure S2 **S6K-dko and food intake.** In spite of their lean phenotype, S6K-dko show higher food intake on standard chow (A) or a high-fat diet (B). BW, body weight; HFD, high-fat diet. Two-way ANOVA *P<0.05, **P<0.01.
(TIF)

Figure S3 **Fatty acids activate S6K1 in vitro in the muscle cell line Sol8.** Linoleic acid increases S6K1 phosphorylation in Sol8 cells in a dose-dependent manner. One way ANOVA **P<0.01.
(TIF)
