# Supplementary figures and images for: Metabolic Control by S6 Kinases Depends on Dietary Lipids
Source: PLoS One. 2012 Mar 7;7(3):e32631. doi: 10.1371/journal.pone.0032631 (PMC3296718; doi:10.1371/journal.pone.0032631)

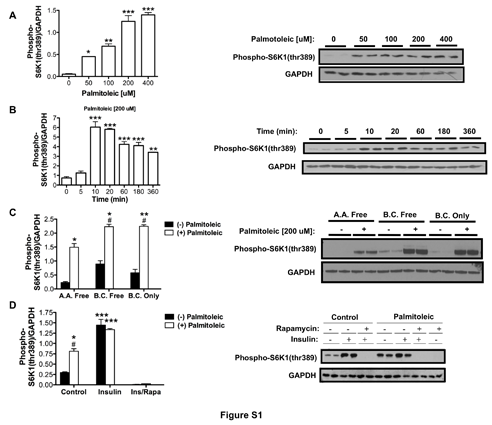

Supplement: Figure S1 — Fatty acids activate S6K1 in vitro in the hypothalamic cell line N-41. Palmitoleic acid increases S6K1 phosphorylation in N-41 cells in a dose-dependent (A) and time-dependent (B) manner. Palmitoleic acid-induced phosphorylation was independent of branched-chain amino acids and amino acids in general (C). S6K1 phosphorylation induced by palmitoleic acid was enhanced by insulin and prevented by rapamycin, suggesting a mechanism involving the mTORC1 pathway (D). A.A., amino acid; B.C., branched-chain amino acid. One way ANOVA *P<0.05, **P<0.01, ***P<0.001; two-tailed t test #P<0.05. (TIF) [file pone.0032631.s001.tif]

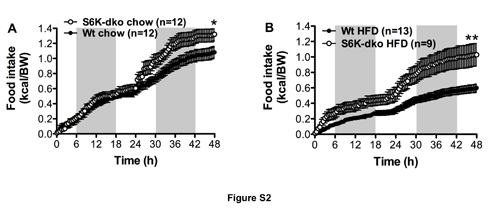

Supplement: Figure S2 — S6K-dko and food intake. In spite of their lean phenotype, S6K-dko show higher food intake on standard chow (A) or a high-fat diet (B). BW, body weight; HFD, high-fat diet. Two-way ANOVA *P<0.05, **P<0.01. (TIF) [file pone.0032631.s002.tif]

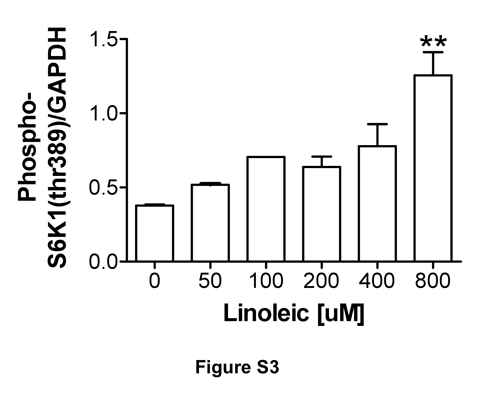

Supplement: Figure S3 — Fatty acids activate S6K1 in vitro in the muscle cell line Sol8. Linoleic acid increases S6K1 phosphorylation in Sol8 cells in a dose-dependent manner. One way ANOVA **P<0.01. (TIF) [file pone.0032631.s003.tif]
